# Supplementary material for: Epidemiology of brucellosis in cattle and dairy farmers of rural Ludhiana, Punjab
Source: PLoS Negl Trop Dis. 2021 Mar 18;15(3):e0009102. doi: 10.1371/journal.pntd.0009102 (PMC8034737; doi:10.1371/journal.pntd.0009102)
Supplement: S1 Table — (DOCX) [file pntd.0009102.s001.docx]

S1 Table Univariate analysis to identify factors associated with Brucella spp. seropositivity at animal-level

| Variable | Frequency (%) | No. Pos (%) | Odds ratio | *P* – value |
| --- | --- | --- | --- | --- |
| CD block |  |  |  |  |
| Jagraon | 515 (28.6%) | 46 (8.9%) | 1 |  |
| East Ludhiana | 487 (27.1%) | 84 (17.2%) | 2.13 (1.46 to 3.14) | <0.001** |
| Payal | 364 (20.2%) | 86 (23.6%) | 3.15 (2.15 to 4.68) | <0.001** |
| Samrala | 432 (24.0%) | 56 (13.0%) | 1.52 (1.01 to 2.30) | 0.05* |
| Total | **1798** | **272** |  |  |
| Species |  |  |  |  |
| Buffalo | 809 (45.4%) | 96 (11.9%) | 1 | - |
| Cow | 972 (54.6%) | 174 (17.9%) | 1.62 (1.24 to 2.13) | <0.001** |
| Total | **1781** | **270** |  |  |
| Breed |  |  |  |  |
| HF | 424 (24.0%) | 102 (11.9%) | 1 |  |
| Buffalo | 806 (45.7%) | 96 (24.1%) | 0.43 (0.31 to 0.58) | <0.001** |
| HF cross | 472 (26.8%) | 63 (13.3%) | 0.49 (0.34 to 0.69) | <0.001 |
| Indigenous | 38 (2.2%) | 5 (13.2%) | 0.48 (0.16 to 1.16) | 0.135* |
| Jersey | 24 (1.4%) | 2 (8.3%) | 0.27 (0.04 to 0.95) | 0.082* |
| Total | **1764** | **268** |  |  |
| Age |  |  |  |  |
| 3 or less | 224 (12.5%) | 34(15.2%) | 1 |  |
| 4 | 604 (33.6%) | 95 (15.7%) | 1.04 (0.69 to 1.61) | 0.846 |
| 5 to 6 | 674 (37.5%) | 94 (13.9%) | 0.91 (0.60 to 1.40) | 0.648 |
| 7 + | 293 (16.3%) | 49 (16.7%) | 1.22 (0.70 to 1.82) | 0.635 |
| Total | **1795** | **272** |  |  |
| Breeding |  |  |  |  |
| AI | 1509 (85.7%) | 220 (14.6%) | 1 |  |
| Natural | 251 (14.3%) | 42 (16.7%) | 1.24 (0.84 to 1.80) | 0.258 |
| Total | **1760** | **262** |  |  |
| Origin |  |  |  |  |
| Bought | 445 (24.9%) | 54 (12.1%) | 1 |  |
| Bred in farm | 1342 (75.1%) | 214 (15.9%) | 1.37 (1.00 to 1.91) | 0.052* |
| Total | **1787** | **268** |  |  |
| Bought |  |  |  |  |
| Farm & other | 301 (69.8%) | 34 (11.3%) | 1 |  |
| Market | 130 (30.2%) | 20 (15.4%) | 1.34 (0.83 to 2.41) | 0.334 |
| Total | **431** | **54** |  |  |
| Bought location |  |  |  |  |
| Another district | 34 (9.6%) | 4 (11.8%) | 1 |  |
| Another village | 297 (83.9%) | 38 (12.8%) | 0.94 (0.27 to 2.57) | 0.92 |
| This village | 23 (6.5%) | 5 (21.7%) | 1.90 (0.60 to 4.09) | 0.23 |
| Total | **354** | **47** |  |  |
| Vaccinated brucellosis |  |  |  |  |
| No | 1770 (98.4%) | 265 (15.0%) | 1 |  |
| Yes | 28 (1.6%) | 7 (25.0%) | 1.89 (0.74 to 4.29) | 0.148* |
| Total | **1798** | **272** |  |  |

*Taken through to multivariate analysis, **significant at the *P* ≤ 0.05 level
