# Supplementary material for: Epidemiology of brucellosis in cattle and dairy farmers of rural Ludhiana, Punjab
Source: PLoS Negl Trop Dis. 2021 Mar 18;15(3):e0009102. doi: 10.1371/journal.pntd.0009102 (PMC8034737; doi:10.1371/journal.pntd.0009102)
Supplement: S2 Table — (DOCX) [file pntd.0009102.s002.docx]

S2 Table Association between demographic variables and Brucella spp. seropositivity in people in direct contact with large ruminants using univariable logistic regression models with village included as a random-effect

| Variable | Frequency (%) | No. Pos (%) | Odds ratio | *P* - value |
| --- | --- | --- | --- | --- |
| Gender |  |  |  |  |
| Female | 394 (69.1%) | 8 (4.6%) | 1 | - |
| Male | 176 (30.9%) | 49 (12.5%) | 3.01 (1.41 to 7.23) | 0.007** |
| Total | **570** | **57** |  |  |
| Age |  |  |  |  |
| Up to 30 | 152 (26.9%) | 19 (12.6%) | 1 | - |
| 31 to 40 | 134 (23.7%) | 18 (14.3%) | 1.22 (0.56 to 2.52) | 0.607* |
| >40 | 279 (49.4%) | 19 (6.8%) | 0.56 (0.27 to 1.16) | 0.116^*^ |
| Total | **565** | **56** |  |  |
| Role on farm |  |  |  |  |
| Family of owner | 317 (56.9%) | 21 (6.6%) | 1 |  |
| Farm worker | 111 (19.9%) | 16 (14.2%) | 2.18 (1.02 to 4.67) | 0.049** |
| Owner | 129 (23.2%) | 18 (13.7%) | 2.37 (1.15 to 4.91) | 0.021** |
| Total | **557** | **55** |  |  |
| Attended school |  |  |  |  |
| No | 147 (26.2%) | 10 (6.8%) | 1 |  |
| Yes | 415 (73.8%) | 45 (10.8%) | 1.71 (0.84 to 3.82) | 0.161* |
| Total | **562** | **55** |  |  |
| State |  |  |  |  |
| Punjab | 470 (82.9%) | 15 (15.5%) | 1 | - |
| Other | 97 (17.1%) | 42 (8.9%) | 0.68 (0.32 to 1.50) | 0.315 |
| Total | **567** | **57** |  |  |

*Taken through to multivariate analysis, **significant at the *P* ≤ 0.05 level
