# Supplementary material for: Epidemiology of brucellosis in cattle and dairy farmers of rural Ludhiana, Punjab
Source: PLoS Negl Trop Dis. 2021 Mar 18;15(3):e0009102. doi: 10.1371/journal.pntd.0009102 (PMC8034737; doi:10.1371/journal.pntd.0009102)
Supplement: S3 Table — (DOCX) [file pntd.0009102.s003.docx]

S3 Table Association between livestock contact and Brucella seropositivity in in people in direct contact with large ruminants using univariable logistic regression models with village included as a random-effect

| Variable | Frequency (%) | No. Pos (%) | Odds ratio | *P* - value |
| --- | --- | --- | --- | --- |
| Milking livestock |  |  |  |  |
| Never + past | 145 (26.3%) | 10 (6.9%) | 1 | - |
| Yes (≤ 12 months) | 406 (73.7%) | 44 (10.8%) | 1.70 (0.82 to 3.81) | 0.172* |
| Total | **551** | **54** |  |  |
| Assisting with calving |  |  |  |  |
| Never + past | 176 (31.8%) | 6 (3.4%) | 1 |  |
| Yes (≤ 12 months) | 377 (68.2%) | 49 (13.0%) | 5.30 (2.28 to 14.6) | <0.001** |
| Total | **553** | **55** |  |  |
| Assisting with abortion in last year |  |  |  |  |
| No | 446 (79.9%) | 28 (6.0%) | 1 |  |
| Yes | 112 (20.1%) | 27 (25.0%) | 5.22 (2.70 to 10.3) | <0.001** |
| Total | **558** | **55** |  |  |
| Contact with goats |  |  |  |  |
| No | 25 (4.4%) | 51 (9.6%) | 1 |  |
| Yes | 532 (95.6%) | 4 (16.0%) | 1.13 (0.26 to 4.05) | 0.862 |
| Total | **557** | **55** |  |  |

*Taken through to multivariate analysis, **significant at the *P* ≤ 0.05 level
