# Supplementary material for: Epidemiology of brucellosis in cattle and dairy farmers of rural Ludhiana, Punjab
Source: PLoS Negl Trop Dis. 2021 Mar 18;15(3):e0009102. doi: 10.1371/journal.pntd.0009102 (PMC8034737; doi:10.1371/journal.pntd.0009102)
Supplement: S4 Table — (DOCX) [file pntd.0009102.s004.docx]

S4 Table Association between dairy consumption and Brucella seropositivity in people in direct contact with livestock using univariable logistic regression models with village included as a random-effect

| Variable | Frequency (%) | No. Pos (%) | Odds ratio | *P* - value |
| --- | --- | --- | --- | --- |
| Consume milk daily |  |  |  |  |
| No | 23 (4.2%) | 1 (4.3%) | 1 | - |
| Yes | 529 (95.8%) | 54 (10.2%) | 0.36 (0.04 to 3.03) | 0.353 |
| Total | **552** | **55** |  |  |
| Boil milk |  |  |  |  |
| Always | 469 (85.1%) | 46 (9.8%) | 1 |  |
| Not always | 82 (14.9%) | 8 (9.8%) | 1.25 (0.49 to 2.96) | 0.620 |
| Total | **551** | **54** |  |  |
| Ever drank raw milk |  |  |  |  |
| No | 453 (80.9%) | 46 (9.6%) | 1 |  |
| Yes | 107 (19.1%) | 11 (10.3%) | 1.24 (0.56 to 2.64) | 0.578 |
| Total | **560** | **57** |  |  |
