# Supplementary material for: Epidemiology of brucellosis in cattle and dairy farmers of rural Ludhiana, Punjab
Source: PLoS Negl Trop Dis. 2021 Mar 18;15(3):e0009102. doi: 10.1371/journal.pntd.0009102 (PMC8034737; doi:10.1371/journal.pntd.0009102)
Supplement: S5 Table — (DOCX) [file pntd.0009102.s005.docx]

S5 Table Association between data from livestock testing and farm questionnaire and Brucella seropositivity in people in direct contact with livestock using univariable logistic regression models with village included as a random-effect

| Variable | Frequency (%) | No. Pos (%) | Odds ratio | *P* - value |
| --- | --- | --- | --- | --- |
| Farm status |  |  |  |  |
| Neg | 357 (62.0%) | 25 (7.0%) | 1 | - |
| Pos | 219 (38.0%) | 29 (13.2%) | 1.85 (0.98 to 3.50) | 0.058 |
| Total | 552 | 54 |  |  |
| Herd size (females) |  |  |  |  |
| Up to 5 | 105 (18.3%) | 7 (6.7%) | 1 | - |
| 6 to 10 | 334 (58.2%) | 37 (11.1%) | 1.90 (0.77 to 5.37) | 0.187 |
| > 10 | 135 (23.5%) | 11 (8.2%) | 1.14 (0.37 to 3.76) | 0.817 |
| Total | 574 | 55 |  |  |
