# Supplementary material for: Epidemiology of brucellosis in cattle and dairy farmers of rural Ludhiana, Punjab
Source: PLoS Negl Trop Dis. 2021 Mar 18;15(3):e0009102. doi: 10.1371/journal.pntd.0009102 (PMC8034737; doi:10.1371/journal.pntd.0009102)
Supplement: S1 Text — (DOCX) [file pntd.0009102.s006.docx]

# Farm study: Questionnaire for farm workers sampled

| General information about the farm worker | |  |
| --- | --- | --- |
| a. Sub-district: □01(EL) □ 02(S) □03(P) □04 (J)  e. Age:_________ f. Gender: □ male □ female | b. Village ID:_________ c. Farm ID:__________  g. First name: _____________________________ | d. Individual ID:________________________  h. Have they ever attended school: □ yes □ no |
| i. Religion: □ Hindu □ Sikh □ Muslim □ Other (specify):__________________________________________ | | k. Highest standard completed: _________ |

| General questions | |  | |
| --- | --- | --- | --- |
| a. What is your role in this farm? | | □ family member □ farm owner □ farm manager □ permanent employee □ occasional/seasonal worker | |
| ***If family member go to question 3*** |  | | |
| b. Are you from the Punjab state? | | □ yes □ no | c. *If no,* which state are you from? |
| d. How many years have you worked in Punjab? | | | e. How long have you worked for this farm? (months/years) *(Delete as appropriate)* |
| f. Where do you reside? | | □ on the farm □ within the village □ another village □ urban area | |

## ACTIVITIES

For each species can you tell us which activities you do or have done in the past, how frequently and where?

| **Species** | **Activity** |  | | | **How often do you participate in this activity?** | | **h. Where do you do these? *(tick all that apply)*** |
| --- | --- | --- | --- | --- | --- | --- | --- |
| **3a.** Cows or buffalo:  □ yes □ no | **b.** Milking: | □ yes (≤12months) | □ never | □ past | □ most days  □ at least once a month | □ at least once a week  □ < monthly (e.) | □ this farm  □ another herd  □ both |
|  | **c.** Assisting with calving: | □ yes (≤12months) | □ never | □ past | **f.** No of times in past 12 months: | |  |
|  | **d.** Assisting with abortion: | □ yes (≤12months) | □ never | □ past | **g.** No of times in past 12 months: | |  |
| **4a.** Goats  □ yes □ no  **b.** Sheep  □ yes □ no | **c.**  Milking: | □ yes (≤12months) | □ never | □ past |  | | |
|  | **d.** Assisting with parturition/abortion: | □ yes (≤12months) | □ never | □ past |  |  |  |
|  | **e.** Slaughtering: | □ yes (≤12months) | □ never | □ past |  |  |  |

**5**. Where applicable, can you tell us the age when you started these activities?

1. Age when started milking cows/buffaloes:____ **b.** Age when started assisting with abortion/calving of cows/buffalo? ____

## CONSUMPTION OF DAIRY PRODUCTS

| **Product** | **A. Frequency** | | **B. Packaged** | | **C. Boiled** | |
| --- | --- | --- | --- | --- | --- | --- |
| **6**. Cow/buffalo milk | □ most days  □ at least once per month  □ never | □ at least once per week  □ less than once per month  □ past | □ always  □ sometimes (<50%)  □ unsure | □ mostly (>50%)  □ never | □ always  □ sometimes (<50%)  □ unsure | □ mostly (>50%)  □ never |
| **7.** Goats’ or sheep milk | □ most days  □ at least once per month  □ never | □ at least once per week  □ less than once per month  □ past |  |  | □ always  □ sometimes (<50%)  □ unsure | □ mostly (>50%)  □ never |

For the following dairy products, can you tell us how frequently you consume them? *(After reading through all the products, go through each and ask whether they are pasteurised or boiled and the location of consumption)*

*If their milk was either ‘always from packaged milk’ or ‘always boiled’ go to question 5*

**8a.** Are there any occasions when you would drink raw milk without boiling it e.g. whilst milking the cow/buffalo:

□ yes □ no *if yes when?:* □ past □ when milking □ **c.** other (specify)*__________________________________________________________________*

## HEALTH

**9.** Please answer the questions about the last 12 months

| **a.** Have you suffered from fever for 2 weeks or longer? | □ Yes □ No □ Unsure | | |  |  | | |
| --- | --- | --- | --- | --- | --- | --- | --- |
| **b.** *If yes* how long ago? |  | Months | | **c.** How long did it last for? |  | | months |
| did you have any additional symptoms with fever such as: | | | | | | | |
| **d.** Intermittent sweating or chills (day or night ) | □ Yes □ No | | □ Unsure | **e.** Lack of energy | | □ Yes □ No □ Unsure | |
| **f.** Body aches | □ Yes □ No | | □ Unsure | **g.** Weight loss | | □ Yes □ No □ Unsure | |
| **h.** Lower back pain | □ Yes □ No | | □ Unsure | **i.** Headaches | | □ Yes □ No □ Unsure | |
| **j.** Joint pains  **k.** If yes which joints? | □ Yes □ No | | □ Unsure | **l.** Abdominal | | □ Yes □ No □ Unsure | |
|  |  |  |  | **m.** testicular swelling | | □ Yes □ No □ Unsure | |
| **n.** Cough | □ Yes □ No | | □ Unsure | **o.** Lack of appetite | | □ Yes □ No □ Unsure | |
| **p.** Did you receive a diagnosis? | □ Yes □ No | | | **q.** What was the diagnosis? | |  | |

**10a.** Have you ever heard of a disease called brucellosis? □ Yes □ No □ Unsure

**10b.** *If yes,* do you think you have ever had brucellosis? □ Yes I am sure I have had it (received medical diagnosis) □ Yes I think I have had it □ No □ Unsure

**Other notes (e.g. antibiotic give, whether they completed course etc.):**
