# Supplementary material for: Epidemiology of brucellosis in cattle and dairy farmers of rural Ludhiana, Punjab
Source: PLoS Negl Trop Dis. 2021 Mar 18;15(3):e0009102. doi: 10.1371/journal.pntd.0009102 (PMC8034737; doi:10.1371/journal.pntd.0009102)
Supplement: Attachment — Submitted filename: PNTD 20-00277 Comments.docx [file pntd.0009102.s007.docx]

**PNTD-D-20-00277 Comments**

Holt et al., described the epidemiology of animal and human brucellosis on dairy farms in rural Ludhiana, Punjab, India. The manuscript is well-written, and results are presented clearly and precisely. The findings from their study suggested a high seroprevalence of the disease in cattle, buffalo, and animal workers on farms in Punjab, as well as the significance of occupational exposure to the disease in this region. Hence, advocating for control and preventive strategies against production losses in animals and *Brucella* infection in animal workers.

While the study and the findings are interesting, there are a few ways in which the manuscript can be improved.

**Abstract:**

Line 6-9: *The study confirmed that brucellosis is endemic at high levels in cattle and buffalo in the study area with 15.1% of large ruminants testing seropositive and approximately a third of dairy farms having at least one animal test seropositive.*

The authors should rephrase this statement. *Confirm* is a strong conclusion and the rate provided may not be reflective of the actual situation simply because the authors based their finding on one serological test. It should be noted that the gold standard for confirming *Brucella* infection is the culture of the bacteria from tissues or bodily fluids of infected animals or humans. However, while culture is 100% specific, it is less sensitive because culture yield has been reported to be inversely correlated with the stage of illness. Nevertheless, when serological tests are employed for the diagnosis of brucellosis, at least two tests are recommended, and a definite diagnosis based on one test is discouraged because of the wide variability in the specificity and sensitivity of the common serological tests. For example, Rose Bengal Test (RBT) is typically used as a screening test and not a confirmatory test because of the caveat mentioned above. RBT has a high sensitivity, which means that some of the animals that are truly positive for brucellosis will be correctly identified, but the low specificity of the test also leads to a high false-positive rate (meaning that those that do not have the disease may be erroneously identified as having the disease). Hence, a recommendation of at least two serological tests for brucellosis diagnosis.

Line 10: italicize *Brucella*

**Author summary:**

Line 4: *This study aimed? to estimate….*

There is a typo here.

Line 7: *The study found high levels in cattle and buffalo in the study area with…..*

High level of what? *Infection/disease/seroprevalence?*

Line 6-8: *The study found high levels in cattle and buffalo in the study area with 15.1% of large ruminants estimated to be infected with Brucella spp. and approximately……*

Infection may not be the right word to use in this context because the statement reads like the animals were actively infected and had observable clinical manifestations of brucellosis or that *Brucella* was cultured from the tissues of the animals. There is no indication that this was the case based on the findings reported in the study.

**Methods:**

Line 21: *….screening for Brucella spp….*

What type of screening? The author should be specific with the type of screening mentioned here. Was it a questionnaire screening about farmers’ knowledge of brucellosis or a serological screening?

Line 26-27: …*Samples were aliquoted into Eppendorf tubes were made and these were then frozen at -20 °C for up to 6 months*….

Typo?

Serological testing:

Line 6: ...*eight medium positive control…*

Authors should clarify or make a note in the discussion section about the rationale of using this type of positive control…was that the kit manufacturer’s recommendation? Why medium positive? Why eight?

For the animal samples, only one serological test (iELISA) was used (please see comment on the abstract above). Why did the authors not use two serological tests in animals as they used for human samples?

Line 13-15: *…Briefly, a sample of serum (0.05 ml) was mixed with 0.05 ml of antigen on a microscope slide to? produce a zone approximately 2 cm in diameter.*

Typo?

Line 16: *…ambient room* temperature. Use room temperature instead?

For quality assurance, the authors should indicate specifically that the samples positive for the antigen provided by the Punjab Vaccine Institute in India were also positive for the antigen from the Animal and Plant Health Agency, UK.

Line 23-25: …*Additionally, all samples reporting positive according to the ELISAs or RBT were tested using the Standard Tube Agglutination Test (SAT) for diagnosis of active disease that may require treatment.*

This was mentioned in the methods but findings from this are not presented in the result section. Were there samples that were positive for the SAT?

Statistical Analysis:

Line 1-2: *Positive samples from livestock were retested and livestock were classified as positive if two positive test results were obtained.*

Retested with what? Same test done twice or another type of serological test? If another, which one?

Line 2-3: *People were classified as seropositive if they tested positive to either the Rose-Bengal test or IgG ELISA.*

Why? Please see the comment above in the abstract section.

Authors should give a brief explanation in the discussion section on the serological diagnostic methods of brucellosis, and the specificity and sensitivity of the tests they used, the rationale for those used in human vs animals and the implications.

Another limitation of the study is that for animals, only milk samples were tested, meaning that male animals which also contribute to prevalence were excluded in the study. *Brucella* is reported to have preference for the reproductive system; therefore, we know males can also harbor and perpetuate the disease particularly during actual mating or servicing of the female animals or when their semen are used for artificial insemination.

**Results:**

Line 3: *Inclusion criteria* like what and what?

*Individual livestock:*

Line 2-4: *…Only 24 (1.3%) animals in four farms were vaccinated against Brucella spp. this was mostly using the reduced dose S19 vaccine via the conjunctival route.*

A common disadvantage of S19 vaccine is its interference with serological diagnosis because of the inability of the tests to differentiate between animals that are infected with field strains from those that were vaccinated with S19. Therefore, animals that were vaccinated with S19 will also test positive with serological tests. This limitation was not accounted for in the results nor was it clarified in the discussion.

*Table 5:*

Is there an agreement between the two serological tests used for human samples? And what is the recommendation for future studies or to investigators that want to do this type of epidemiological study in other endemic countries?

**Discussion:**

Line 4: *The study confirmed that brucellosis is endemic at high levels in cattle and buffalo of rural Ludhiana district in Punjab*. If both serological tests were used and most of the animals were positive, then yes. If not, a confirmatory test like culture or at least two serological tests are required to make a definite conclusion to avoid the bias of a high false-positive rate. Additionally, the limitation of the serological testing of animals on farms that vaccinated with S19 should be addressed and reflected in the results (please see the comment above).

Paragraph 4 Line 1: *As people are primarily infected via animal sources, control in livestock (primarily ruminants) is key to reducing the public health impact of the disease.*

Results from this study indicate that human prevalence is only high in those in close contact with animals (e.g. farmers, livestock keepers etc.), hence the disease in this region appears to be more restricted to occupational exposure. While this study highlights the significance of occupational exposure to the disease, it doesn’t explain the prevalence in the general population especially because the disease does not appear to be transmitted through milk consumption which will pose a significant threat to the general population. This is because the authors reported that most people in the region in which the study was conducted consume milk that are either boiled or pasteurized, therefore, it seem that the general population that do not have any contact with animals or consume raw milk may not be at significant risk because humans are regarded as dead end hosts (human to human transmission of the brucellosis is rare). Therefore, the authors need to make stronger claims or speculate other ways in which the general population that have no contact whatsoever with animals may be at significant risk of *Brucella* infection.
