# Supplementary material for: Epidemiology of brucellosis in cattle and dairy farmers of rural Ludhiana, Punjab
Source: PLoS Negl Trop Dis. 2021 Mar 18;15(3):e0009102. doi: 10.1371/journal.pntd.0009102 (PMC8034737; doi:10.1371/journal.pntd.0009102)
Supplement: Attachment — Submitted filename: PNTD 20-00277 Comments Final.docx [file pntd.0009102.s008.docx]

**PNTD-D-20-00277 Comments**

Reviewer #1:

This study was conducted to estimate the prevalence of brucellosis in cattle, buffalo and in-contact humans and to identify the risk factors for seropositivity. The manuscript has some novel data about brucellosis epidemiology in India. The authors should be commended for using a robust design for the study, in particular, for using probabilistic sampling methods to select villages and animals. However, the casual approach to data analysis and writing the manuscript was disappointing and could have been improved. The manuscript has many grammatical errors, particularly, in the first two sections. I have listed some of the issues above, but the authors should read the manuscript thoroughly before resubmission. The manuscript should be of interest to the journal audience, but the authors would have to rebuild most of the models and thoroughly revise the manuscript before it could be accepted for publication.

We would like to thank the reviewer for taking the time to revise the manuscript.

The manuscript has been read thoroughly for grammatical errors and we have improved the manuscript based on all the reviewers’ comments. The statistical analysis has been revisited and we have replied to the reviewers concerns with this below. We hope the manuscript is now improved to the reviewers’ satisfaction.

Abstract
Change “The aim of this study is to” to “The aim of this study was to”

Changed (line 24).

Author summary

Reword this sentence: “This study to estimate the frequency of exposure to the bacteria (Brucella spp.) in dairy animals in Ludhiana district in Punjab State of India and persons in contact with these livestock either through their occupation or household.”

This has been reworded (lines 41:45)

*‘This study to aimed estimate the frequency of exposure to the bacteria (Brucella spp.) in cattle and buffalo in Ludhiana district, Punjab State, India. In addition, persons in contact with these livestock, either through their occupation or household, were also screened for Brucella spp. antibodies and the data used to identify factors increasing their risk of exposure.’*

Introduction
Replace comma with a period: India is currently the world’s leading milk producer. This study was conducted…

Changed (line 53)

Reword: produces the most cattle and buffalo’ milk…

This has been reworded (lines 53:55)

*‘India is currently the world’s leading milk producer; this study was conducted in Punjab State which has the highest per capita milk production of all the Indian States (937g per person per day).’*

Reword or split: Endemic and emerging livestock diseases pose a threat to the milk industry due to reduced productivity of livestock (direct impact), trade barriers and, if a milkborne zoonosis, posing a threat to consumer health.

This has been reworded (lines 57:59)

*Endemic and emerging livestock diseases pose a threat to the milk industry due to reduced productivity of livestock (direct impact) and trade barriers (indirect). In addition, milkborne pathogens pose a threat to consumer health.*

Reference has been changed (line 63):

*In livestock, the disease reportedly causes economic losses to farmers through abortions and subsequently decreased milk yield (Abernethy et al., 2011; Xavier et al., 2009).*

Reword or split: However, many of the previous studies have not used
probabilistic sampling therefore the estimates may be biased, an example of `one study where sampling was unbiased was conducted over 10-years ago and reported a lower animal-level seroprevalence estimate.

Changed (lines 70:72):

*However, many of the previous studies have not used probabilistic sampling therefore the estimates may be biased. One study where sampling was unbiased was conducted over 10-years ago and reported a lower animal-level seroprevalence estimate (11.2%) (Dhand et al., 2005).*

Reword or split: Incidence of the disease in humans relates to the frequency of brucellosis in the local livestock population it is an occupational zoonosis for farmers and veterinarians

Reworded and moved (lines 76:78):

*‘Brucellosis is often cited as one of the world’s most common bacterial zoonosis and is transmitted to humans via the oral, respiratory, or conjunctival routes either via direct contact or consumption of unpasteurised milk and dairy products (Crawford et al., 1990; Pappas et al., 2006). Therefore, Incidence of the disease in humans relates to the frequency of brucellosis in the local livestock population and it is an occupational zoonosis for farmers and veterinarians (Musallam et al., 2016; Proch et al., 2018).’*

Reword or combine: Only one sample was positive by RBT but negative by IgG ELISA. Whereas 40 samples were positive IgG ELISA and negative by RBT.

Reworded (line 298):

*Only one sample was positive by RBT but negative by IgG ELISA, whereas 40 samples were positive by IgG ELISA and negative by RBT.*

Split into two sentences: Only 24 (1.3%) animals in four farms were vaccinated against Brucella spp. this was mostly using the reduced

Split (lines 275:277) :

*‘Only 24 (1.3%) animals in four farms were vaccinated against Brucella spp. These animals were vaccinated using the reduced dose S19 vaccine via the conjunctival route.’*

Reword or combine: The majority of the participants were male (69.1%) and over 20 (92.4%). With family members accounting for the majority of people in occupational contact with cattle on dairy farms (56.9%), with around owners accounting for 23.2% and employees accounting for 19.9%.

These lines have been deleted.

Reviewer #1 (analysis):

The creation of variables for analysis is unplanned. Many variables could have been merged to reduce the number of variables. The list/number of variables used to build models should be explicitly presented.

We apologise for not making the rationale clearer – we have now included a causal diagram for risk factors and this diagram (Figure 1), combined with the univariate tables states the variables used to build the models.

Be consistent with the use of terms infected, positive and seropositive. Avoid using them interchangeably. Please double check everywhere in the manuscript.

Checked: use seropositive throughout (positive for antibodies). Unless referring to a positive classification on a given test e.g. ‘positive by RBT, positive by IgG iELISA’ etc.

East Ludhiana has the largest population: of animals or humans?

Clarified (line 125):

*‘East Ludhiana has the largest human population’*

It is not clear why eight herds were selected in each village.

This was done with the aim of meeting the target sample size: 411/60 = 6.85. Rounded up to 8 as some villages would not have enough eligible herds. This has been made clearer (lines 129: 130)

*‘Within these Tehsils, 60 villages were randomly selected using sampling probability proportional to human size (Census of India, 2011).*

*In order to meet the required sample size. eight dairy farms per village were selected using simple random sampling. Lists obtained from the designated veterinary officer or pharmacist of each village were used as a sampling frame. If eight of fewer eligible farms were present in the study area then all farms were approached for inclusion in the study.’*

Serological testing: Do the serological tests used in the study detect antibodies against Brucella abortus or any Brucella spp?

The serological tests detect antibodies against smooth lipopolysaccharide shared between smooth strains of *Brucella* (*B. melitensis, B. abortus, B suis*).

The ELISA used for livestock (BRUCELISA) is recommended an indirect ELISA assay for the detection of antibodies against *Brucella abortus* in bovine milk samples. However, cross-reactions with other smooth strains of *Brucella* spp. can occur.

The following has been reworded (lines: 169: 171)

*Cattle and buffalo milk samples were screened for Brucella spp. using a commercial indirect milk ELISA developed by the OIE brucellosis reference laboratory at the Animal and Plant Health Agency, UK (BRUCELISA®). The assay detects IgG antibodies to smooth Brucella strains in bovine milk samples.*

The following has been reworded (lines: 195:197)

*‘Both serological tests detect antibodies against the smooth lipopolysaccharide shared between the smooth strains of Brucella spp. (B. abortus, B. melitensis and B. suis). Testing for human sera was performed in the School of Public Health and Zoonosis, GADVASU.’*

No results for SAT testing have been provided. Please clarify.

The SAT results have been added to the paper (they were previously removed to reduce the size of the manuscript) (Table 7):

| **Result** | ***N*** | **RBT +** | **IgG ELISA +** | **Symptoms?** |
| --- | --- | --- | --- | --- |
| 1/80 | 16 | 15 | 16 | 2 (1 fever, 1 joint pain) |
| 1/160 | 15 | 14 | 15 | 2 (1 fever, 1 joint pain) |
| 1/320 | 13 | 13 | 13 | 2 (1 fever, 1 joint pain) |

The following has been added to the text:

Lines (300:303):

*‘Of the 57 people that were classified as seropositive, 13 (22.8%) produced SAT titers of 1/320 or higher. All of these were RBT positive, however, only 2 mentioned symptoms in the last 12 months’*

Lines (203:207):

*Persons with a SAT titres of 1/320 or higher received a phone-call from a clinician at GADVASU who works with farmers and veterinarians to diagnose and treat brucellosis in the study area for further follow-up/treatment.*

It is not clear if the same samples were retested or repeated samples were obtained from animals. The interpretation of results from these two scenarios would be different. Three sentences from different sections with different meanings are copied below.

These sentences have been reworded as follows:

“All seropositive samples and a proportion of randomly selected negative samples were retested using the same procedure.”

Lines 179:181

*‘All milk samples which tested seropositive and a proportion of randomly selected negative samples were retested using the same procedure.’*

“Positive samples from livestock were retested and livestock were classified as positive if two positive test results were obtained. People were classified as seropositive if they tested positive to either the Rose-Bengal test or IgG ELISA.”

Lines 209:213

*‘Seropositive samples from livestock were retested with the same BRUCELISA® test and livestock were classified as positive if two seropositive test results were obtained. People were classified as seropositive if they tested positive to either RBT or IgG ELISA. Farms were classified as positive if at least one animal within the farm was classified seropositive for Brucella spp. after serial testing with the iELISA.’*

“Of the 1854 cattle and buffalo sampled, 280 tested positive on two consecutive milk ELISA’s,”

Changed - final numbers have changed after performing some additional quality assurance (Lines 248:250):

*Of the 1802 milk sample collected from cattle and buffalo, 312 initially gave a positive result and 272 produced two consecutive positive results using the commercial IgG ELISA (BRUCELISA®).*

Change the sentence “Univariate analysis was performed” to “Univariate logistic regression analyses were performed”

Changed (Line 214).

Similarly, specify the model used for multivariable analyses: generalised linear mixed models (GLMM).

Done (Line 225)

*‘Three generalised linear mixed models (GLMM) were then used to identify risk factors for i) individual animals (with farm and village included as a random effect), ii) farms (with village included as a random effect) and iii) persons in direct contact with livestock (with village included as a random effect) being classified as seropositive, controlling for potential confounders’*

ICC: ICC can be calculated from GLMM, so you don't need to conduct ANOVA. Please refer to Vet Epi Research by Ian Dohoo, if not sure.

This has been corrected and GLMM ICC’s are now presented (there were some errors in the previous calculations, thanks to the reviewer for pointing this out):

Line 270:272

*The adjusted ICC of the farm model was 0.11, suggesting that 11% of the variance can be accounted for by the clustering of people within village.*

Line 285:286

*The adjusted ICC for individual livestock was 0.164 for village and 0.005 for farm.*

Line 315:317

*The adjusted ICC of the model for persons in direct contact with livestock was 0.204.*

“Age was included as an a priori confounder” It is not clear if the human or animal age was included. Also, it is not clear in which model age was included as a confounder.

Included in both (and added to the text) Lines 281 & 335

Were the assumptions of the model evaluated? If yes, how? Were the assumptions met

The model assumes the following

- The random effects are assumed to be normally distributed. This one is not to be verified as we have made such assumption when fitting the model.
- Ensured there was an adequate sample size. Checked minimum of 10 cases with the least frequent outcome for each independent variable taken forward to multivariate.
- The data should follow a Bernoulli/binomial distribution; observations are independent after accounting for the clustering within the village
- Linear increase in log odds assumption was checked for continuous varies and we also ensured there is not extreme value (see later)
- No collinearity among predictors (see below)
- Interaction was checked for (see below)

This has now been made clearer in the statistical analysis section and in the causal diagram.

Did you test interactions between significant variables in the model? It became apparent from the last model that the interactions were not tested. Please justify.

Risk factors for people testing seropositive:

The only interaction between model variables that was considered to be biologically plausible was the interaction between calving/abortion and presence of positive animal in the herd (see conceptual diagram for risk factors). This was tested for, however, the very few observations in some categories (positive people that did not assist with calving/abortion in both positive and negative farms) was insufficient for inclusion of an interaction term between these variables. Therefore, a new variable was created combining people that ‘do not assist with calving/abortion’ into one category.

Animal-level:

It was considered that species could potentially interact with other variables, this was checked however, interaction was not present.

Farm-level

Instead of having ‘no of cows’ the model was also fitted with the binary variables cows yes/no and interaction with the disinfection variable. No interaction was present. Interaction between disinfection and whether animals were separated at calving was also checked and not present.

See conceptual diagram and methods for further information.

Did you test for potential collinearity between explanatory variables? If yes, how?

See lines (236: 237)

*‘Each model was checked for multicollinearity by calculating the Variance Inflation Factor (VIF) using the R package ‘performance’; VIF values greater than 10 were considered non-tolerable’*

Example output:

Reviewer #1: ALL TABLES:
- Please report overall p-values for each variable. These p-values should be used to make decisions about whether or not a variable is significant, instead of the p-values currently presented.

Lines 229:234

*‘Upon addition of each variable, likelihood ratio tests were performed comparing the fit of the new model with the previous. Variables were retained if there was significant evidence (P-value ≤ 0.0.5) against the null hypothesis that the simpler model was better and/or their addition changed (+/-) the OR for the association between another variable and Brucella seropositivity by ≥ 10% (possible confounding).’*

- Odds ratio for the reference category should be 1. Please replace ‘-‘ with 1 in all tables.

Replaced.

Table 1
- Not sure why cows and buffalo are two different variables. The variable herd type adequately captures this information. retain herd type and delete the other two variables.

Deleted.

- What is the reason for testing the variables cow and buffalo calves? Justify or exclude.

*As well as investigating risk factors for Brucella exposure – the univariate tables give a description of the population in the study e.g. herd demographics. However, as it is not relevant of a risk factor we have excluded and the study data is available on request.*

- Is there any reason for including number of cows and buffaloes separately, in addition to the variable total herd size? Justify or exclude (i.e. just retain total herd size).

It has been suggested that the susceptibility to *Brucella* infection may differ between cattle and buffalo and there are also differences in how the species managed. These variables were then investigated separately and together (total herd size). As increasing herd size and total number of cows increased the odds of the farms testing seropositive. As these variables exhibited collinearity only one was taken forward to the multivariate analysis. As increasing total number of buffaloes in the herd **decreased** the odds of testing seropositive (likely due to confounding – herds less likely to have cows), it was felt that including total number of cows was more appropriate than total herd size.

We were conscious of not having too much text in the document explaining why each variable was retained/rejected. However, this is now explained in Figure 1.

Table 2
What is LR? Please explain the abbreviation in a footnote to the table.

This was large ruminants but has now been changed to livestock.

Insemination cows, insemination buffalo, natural service: why are there three variables instead of one? The variable should be ‘type of insemination: natural or AI’

These variables were not mutually exclusive. They depend on whether the farm has cattle and buffalo or both. In addition, some farms used AI for cows and natural service for buffaloes.

Table 3
- Please see my comment about overall p-values above.

- Why are the odds ratios for Sub-district very different from those of univariable results? For example, the odds of Samarala are nearly double than that of East Ludhiana in Table 1 and nearly half of East Ludhiana in Table 3. Please double check to make sure that you are using the same categories as in Table 1. Note that the odds ratios can change after the addition of other variables in the model, but such a drastic change needs to be investigated (e.g. the variables in the final model may be collinear).

We would like to thank the reviewer for spotting this – the odds ratios were the wrong way around it Table 1. This has been corrected.

Also, the herd prevalence estimates in Table S2 do not seem to align with the estimates in Table 1. Please review these data and results.

Table 1 is univariate analysis for **herds testing seropositive for Brucella.** S1 is univariate analysis for **animals testing seropositive for Brucella** i.e. results are animal level. This has been made clearer in Table legends.

- I assume that the ‘number of cows’ variable was used as a numeric variable. Did you test whether its association with the logit of the outcome linear?

Likelihood ratio tests preferred the model where number of cows was linear.

- Add the reference category for the last variable in Table 3.

Done

Table S3:
- Why are there two variables for ‘role on farm’ and religion?
- It doesn’t seem appropriate to combine Muslims with Hindus just because their numbers are small. The proportions seropositive were actually similar in Sikh and Hindus, so you are going to make incorrect conclusions by clubbing Hindus with Muslims.

The two variables were to show how the categories of the variables in the questionnaire and how categories had been collapsed for the analysis. However, we have now just presented the categories used for the analysis. We agree with the reviewer and as religion itself is not a biologically plausible risk factor and more related to the role on the farm we have dropped it from the analysis.

Table S4:
- It is not clear why odds ratios and p-values have been suppressed for some variables.
- Why are there two variables for each of the following variables: ‘milking LR’, ‘assisting with calving’, ‘assisting with abortion?

Odds ratios and p-values were not presented where the number of observations in at least one of the categories were too few to investigate statistical associations. However, these variables have now been dropped from the tables.

-
Table 4
- Looks like age is used as a numeric variable here. Did you test the assumption of linearity for age?

We apologies as the assumption of linearity for age is not met in this case (however, it is met in the livestock model). Age is now entered into the model as categorical.

- In contrast, in Table S4, age was included as a categorial variable. Please be consistent. Why was age categorised for univariable analyses and then used as numeric for multivariable analyses?

This is now consistent.

Table 5 is unnecessary. Just briefly present the results in the text.

Deleted

Table 6
- Combining never with the past category is not advisable.

There were too few observations for ‘past’ in the dataset for it to remain a standalone category. Although we agree with the reviewer that collapsing of never and past is not ideal, the proportions seropositive for ‘past’ are much closer to ‘never’ than ‘current’. Therefore, we felt by combining ‘past’ with ‘current’ we would make incorrect conclusions (as the reviewer felt combining Muslim and Hindus would lead to incorrect conclusions).

- Which variables were tested in this model?

For all multivariate analyses, the variables tested are marked in the univariate tables and a footnote informs the reader which were taken through to the multivariate analysis. A causal diagram is now included.

- Why not test if the interactions were significant, instead of arbitrarily combining variables? Delete Tables 8 and 9 and include the interaction term in Table 6 itself.

Interaction was tested for, however, the very few observations in some categories (positive people that did not assist with calving/abortion in both positive and negative farms) was insufficient for inclusion of an interaction term between these variables and the model did not converge. Therefore, a new variable was created combining people that ‘do not assist with calving/abortion’ into one category. However, only the final multivariate model is now included in the text:

Lines 321:333

*The results of the multivariate model are presented in Table 8. Assisting with calving and assisting with abortion were combined in to a new variable (“assisting with calving and/or abortion in the last 12 months”) as GLMM did not converge when included separately in the final model. In the univariate analysis, people in direct contact with seropositive cattle and buffalo had 1.85 (0.98 to 3.50) times the odds of testing seropositive for Brucella spp. compared to those that did not. A biologically plausible interaction between farm status and association between assisting with calving/abortion and brucellosis status was investigated. However, only 5 people (5.7%) that did not assist with calving/abortion were seropositive (1 from a seropositive farm and 1 from a seronegative farm). As there were two few observations to include an interaction term between these variables, a new variable with three categories was created: i) Have not assisted with calving or abortion in the past 12 months, ii) assisted with calving and/or abortion in the last 12 months and from a farm classified as seronegative and iii) assisted with calving and/or abortion in the last 12 months and from a farm classified as seropositive.*

Section 2.1.2
- Please report ICC values for other models.

Done, see response to previous comment.

Table 6: Can you please clarify which variables were included in the model presented in Table 6? Were all the variables in tables from S3 to S5 tested in this model?

For all multivariate analyses, the variables tested are marked in the univariate tables and a footnote informs the reader which were taken through to the multivariate analysis. If these tables are in the supplementary then it is mentioned in the text also:

Lines (279:280)

*CD block, species, breed, origin and whether or not the animal was vaccinated against Brucella spp. were taken forward to the multivariate analysis.*

Lines (309:310)

*CD block, gender, role on the farm, level of schooling and activities in the farm (milking; assisting with calving; assisting with abortion) were taken forward to the multivariate analysis,*

“Angandwadi workers”: Briefly describe who they are. Are they healthcare workers or medical practitioners?

Anganwadi workers are rural healthcare workers primarily responsible for child and maternal health. Changed to ‘rural healthcare workers’

Discussion, second last paragraph: Please define PPE.

Done

Discussion, last paragraph: “Given the absence of PPE use in the current survey”. I might have missed but didn't notice this finding in the current study. Please clarify.

Clarified as follows (lines 484:499)

*As the primary risk factor was assisting with calving/abortion, public health campaigns should also include recommendations regarding the use of personal protective equipment (PPE) when assisting with calving. The final questionnaire for persons in direct contact with livestock did not contain questions on the use of PPE as results of the pilot surveys and experience of the field team suggest it is extremely rare for PPE to be worn whilst milking or assisting with parturition. This was observed during fieldwork and lack of PPE has been described in other brucellosis endemic settings (Holt et al., 2011; Mangalgi et al., 2016; Musallam et al., 2015). Therefore, consideration as to whether this is a realistic strategy is needed. In addition to reducing the risk of exposure to the individual, appropriate hygiene and management of animals during calving can also reduce within-herd transmission of the disease in livestock. Clear messages backed by scientific evidence as to what farmers can do to protect themselves, their workers and their livestock from brucellosis are needed. These should be informed by assessments considering cost, feasibility and acceptability. Veterinary officers working in villages and the university extension services, which hold farmers’ fayres and veterinary camps, are best placed to disseminate this knowledge. However, it is important that smallholders also receive these messages.*

Reviewer #2:

Holt et al., described the epidemiology of animal and human brucellosis on dairy farms in rural Ludhiana, Punjab, India. The manuscript is well-written, and results are presented clearly and precisely. The findings from their study suggested a high seroprevalence of the disease in cattle, buffalo, and animal workers on farms in Punjab, as well as the significance of occupational exposure to the disease in this region. Hence, advocating for control and preventive strategies against production losses in animals and *Brucella* infection in animal workers.

While the study and the findings are interesting, there are a few ways in which the manuscript can be improved.

We would like to thank the reviewer for their positive responses on the manuscript, after taking their comments into account we feel the manuscript is much improved*.*

**Abstract:**

Line 6-9: *The study confirmed that brucellosis is endemic at high levels in cattle and buffalo in the study area with 15.1% of large ruminants testing seropositive and approximately a third of dairy farms having at least one animal test seropositive.*

The authors should rephrase this statement. *Confirm* is a strong conclusion and the rate provided may not be reflective of the actual situation simply because the authors based their finding on one serological test. It should be noted that the gold standard for confirming *Brucella* infection is the culture of the bacteria from tissues or bodily fluids of infected animals or humans. However, while culture is 100% specific, it is less sensitive because culture yield has been reported to be inversely correlated with the stage of illness. Nevertheless, when serological tests are employed for the diagnosis of brucellosis, at least two tests are recommended, and a definite diagnosis based on one test is discouraged because of the wide variability in the specificity and sensitivity of the common serological tests. For example, Rose Bengal Test (RBT) is typically used as a screening test and not a confirmatory test because of the caveat mentioned above. RBT has a high sensitivity, which means that some of the animals that are truly positive for brucellosis will be correctly identified, but the low specificity of the test also leads to a high false-positive rate (meaning that those that do not have the disease may be erroneously identified as having the disease). Hence, a recommendation of at least two serological tests for brucellosis diagnosis.

We agree with the reviewer that confirm is a misleading term, therefore we have changed the text as follows (lines 27:30):

*‘The study suggests that brucellosis is endemic at high levels in cattle and buffalo in the study area with 15.1% of large ruminants testing seropositive and approximately a third of dairy farms having at least one animal test seropositive.’*

See also response to later comment regarding this.

Line 10: italicize *Brucella*

Done.

**Author summary:**

Line 4: *This study aimed? to estimate….*

There is a typo here.

This has been changed

Line 7: *The study found high levels in cattle and buffalo in the study area with…..*

High level of what? *Infection/disease/seroprevalence?*

Line 6-8: *The study found high levels in cattle and buffalo in the study area with 15.1% of large ruminants estimated to be infected with Brucella spp. and approximately……*

Infection may not be the right word to use in this context because the statement reads like the animals were actively infected and had observable clinical manifestations of brucellosis or that *Brucella* was cultured from the tissues of the animals. There is no indication that this was the case based on the findings reported in the study.

This has been reworded as follows (lines 45:47):

*“The study found high levels of exposure in cattle and buffalo in the study area with 15.1% of large ruminants testing positive for antibodies to Brucella spp. and approximately a third of dairy farms having at least one animal testing positive.”*

**Methods:**

Line 21: *….screening for Brucella spp….*

What type of screening? The author should be specific with the type of screening mentioned here. Was it a questionnaire screening about farmers’ knowledge of brucellosis or a serological screening?

This has been corrected as follows (lines 136:138):

*‘Within selected farms, all people present involved with livestock husbandry (mostly farm owners, employees and family members) were offered serological screening for Brucella spp.’*

Line 26-27: …*Samples were aliquoted into Eppendorf tubes were made and these were then frozen at -20 °C for up to 6 months*….

Typo?

This has been corrected (142:144):

*‘Samples were aliquoted into Eppendorf tubes and these were then frozen at -20 °C for up to 6 months before de-frosting at room temperature for testing.’*

Serological testing:

Line 6: ...*eight medium positive control…*

Authors should clarify or make a note in the discussion section about the rationale of using this type of positive control…was that the kit manufacturer’s recommendation? Why medium positive? Why eight?

The commercial kit currently recommends that the cut-off is 10% of the positive control. However,

the medium positive (rather than a strong positive) was used so that the positive/negative cut-off can be defined more robustly/reproducibly (compared to use of a strong positive control). This is because the medium positive is closer on the dose-response curve to that cut-off than a strong positive control. Thus, potential inter-plate variation due to any fluctuation in the shape of the dose-response curve is minimized. Using the medium cut-off is now being trialed in the UK surveillance programs for brucellosis.

Eight control samples were used so that a robust average could be calculated and the variation between the 8 replicates can be used to monitor intra-plate variation. This helps to maintain high quality (as plates showing high variation, in accordance to predefined criteria, can be discarded and samples retested). Although eight has been removed from the text as this is slightly arbitrary – just that columns G and H were reserved for control/blank wells. As the medium control was used as the cut-off sufficient replicates of this control were therefore APHA scientists advise to make the whole of column G medium positive.

**Note:** The methods for screening livestock was developed in conjunction with senior scientists at the OIE reference for laboratory at the Animal and Plant Health Agency in the UK. Training was provided to researchers conducting the testing by APHA scientists.

This has been clarified (lines 173:178):

*A positive/negative cut-off was calculated as 50% of the mean of the optical density (OD) of the medium positive control wells. The medium positive was used so that the positive/negative cut-off can be defined more robustly as it is closer on the dose-response curve to that cut-off than a strong positive control. Thus, potential inter-plate variation due to any fluctuation in the shape of the dose-response curve is minimised (Senior Scientist, APHA)*

For the animal samples, only one serological test (iELISA) was used (please see comment on the abstract above). Why did the authors not use two serological tests in animals as they used for human samples?

Only milk samples were collected from cattle and buffalo as milk samples were more acceptable to farmers than taking blood samples from their livestock, also a lack of facilities to restrain the animals made this safer for fieldstaff. As serum samples were not collected from all livestock only the milk iELISA was performed.

As the study was a population survey aimed at generating seroprevalence estimates and the aim was not to provide definitive diagnostics for individual animals, we acknowledge that there may have been misclassification of some animals. However, the iELISA is the same used in the brucellosis surveillance program in the UK and is produced by an official OIE reference laboratory. The specificity is estimated to be 99.93% (validated against 4146 samples from animals in the UK that are brucellosis free). The milk ELISA comfortably conforms to the analytical sensitivity requirements of the OIE (as per this chapter [https://www.oie.int/fileadmin/Home/eng/Health_standards/tahm/3.01.04_BRUCELLOSIS.pdf section 2.9.1.1](https://www.oie.int/fileadmin/Home/eng/Health_standards/tahm/3.01.04_BRUCELLOSIS.pdf%20section%202.9.1.1)) and the European Union (Council Directive 64/432/EEC).

For the reviewer’s information, another aim of the project was to assess correlation serum was collected from a sub-set of these livestock and screened using RBT antigen from APHA (results being prepared for publication elsewhere). The milk ELISA and RBT showed very high correlation – results shown here (Inc are those samples producing discordant results with milk ELISA):

|  | | **RBT** | |
| --- | --- | --- | --- |
|  |  | **-** | **+** |
| **sLPS iELSA (milk)** | **-** | 388 | 0 |
|  | **inc** | 1 | 7 |
|  | **+** | 2 | 79 |

See response to later comment for additions to the manuscript.

Line 13-15: *…Briefly, a sample of serum (0.05 ml) was mixed with 0.05 ml of antigen on a microscope slide to? produce a zone approximately 2 cm in diameter.*

Typo?

This has been corrected.

Line 16: *…ambient room* temperature. Use room temperature instead?

Done.

For quality assurance, the authors should indicate specifically that the samples positive for the antigen provided by the Punjab Vaccine Institute in India were also positive for the antigen from the Animal and Plant Health Agency, UK.

Added: *‘These samples showed perfect correlation’*

Line 23-25: …*Additionally, all samples reporting positive according to the ELISAs or RBT were tested using the Standard Tube Agglutination Test (SAT) for diagnosis of active disease that may require treatment.*

This was mentioned in the methods but findings from this are not presented in the result section. Were there samples that were positive for the SAT?

The SAT results have been added to the paper (they were previously removed to reduce the size of the manuscript):

| **Result** | ***N*** | **RBT +** | **IgG ELISA +** | **Symptoms?** |
| --- | --- | --- | --- | --- |
| 1/80 | 16 | 15 | 16 | 2 (1 fever, 1 joint pain) |
| 1/160 | 15 | 14 | 15 | 2 (1 fever, 1 joint pain) |
| 1/320 | 13 | 13 | 13 | 2 (1 fever, 1 joint pain) |

The following has been added to the text (Lines 300:303):

*‘Of the 57 people that were classified as seropositive, 13 (22.8%) produced SAT titres of 1/320 or higher. All of these were RBT positive, however, only 2 mentioned symptoms in the last 12 months’*

Statistical Analysis:

Line 1-2: *Positive samples from livestock were retested and livestock were classified as positive if two positive test results were obtained.*

Retested with what? Same test done twice or another type of serological test? If another, which one?

This has been clarified (lines 209:210):

*Seropositive samples from livestock were retested with the same BRUCELISA® test and livestock were classified as positive if two seropositive test results were obtained.*

Line 2-3: *People were classified as seropositive if they tested positive to either the Rose-Bengal test or IgG ELISA.*

Why? Please see the comment above in the abstract section.

Both methods are complementary. The RBT can detect three families of brucella antibodies (IgM, IgA and IgG) against surface lipopolysaccharide antigens, whereas ELISAs mostly use cytoplasmic proteins as antigens to detect a specific antibody family (in this case IgG, which last the longest following priming of the immune system by the pathogen. Testing has been discussed in more detail (see next comment).

Authors should give a brief explanation in the discussion section on the serological diagnostic methods of brucellosis, and the specificity and sensitivity of the tests they used, the rationale for those used in human vs animals and the implications.

The estimated sensitivity and specificity of the milk ELISA is given in Table 1. This test is developed by the OIE brucellosis reference laboratory at the APHA. The milk ELISA the specificity is 99.93% (validated against 4146 samples from animals in the UK that are brucellosis free). The milk ELISA comfortably conforms to the analytical sensitivity requirements of the OIE (as per this chapter [https://www.oie.int/fileadmin/Home/eng/Health_standards/tahm/3.01.04_BRUCELLOSIS.pdf section 2.9.1.1](https://www.oie.int/fileadmin/Home/eng/Health_standards/tahm/3.01.04_BRUCELLOSIS.pdf%20section%202.9.1.1)) and the European Union (Council Directive 64/432/EEC).

This has been added to the discussion:

Information on the human ELISA has been added to the methods (line 197:200).

*‘The Demeditec IgG iELISA (used in the serological testing of humans for prevalence of infection) was validated by the manufacturer though parallel testing against a CE-marked equivalent ELISA product. This study gave a value of 100% specificity (n=88) and 100% sensitivity (n=9).’*

Further discussion on issues with human diagnostic issues is also provided (see response to later comment).

Another limitation of the study is that for animals, only milk samples were tested, meaning that male animals which also contribute to prevalence were excluded in the study. *Brucella* is reported to have preference for the reproductive system; therefore, we know males can also harbor and perpetuate the disease particularly during actual mating or servicing of the female animals or when their semen are used for artificial insemination.

We agree with the reviewer. However, it was rare that farms had male animals present and where present, these were mostly local breeds used for traction. However, artificial vs. natural insemination was investigated as a risk factor for the disease. By including only lactating animals we acknowledge that there are cattle and buffalo populations missed in the survey.

The following has been added to the discussion (lines 404:406):

*Only lactating animals were sampled, therefore male animals (which may play a role in disease transmission) were not tested, however, most farms were not keeping male livestock for breeding.*

**Results:**

Line 3: *Inclusion criteria* like what and what?

Clarified (line 244):

*‘The cross-sectional study included 413 farms in 58 villages (two of the villages did not have farms meeting the criteria - four adult cattle and/or buffalo).’*

*Individual livestock:*

Line 2-4: *…Only 24 (1.3%) animals in four farms were vaccinated against Brucella spp. this was mostly using the reduced dose S19 vaccine via the conjunctival route.*

A common disadvantage of S19 vaccine is its interference with serological diagnosis because of the inability of the tests to differentiate between animals that are infected with field strains from those that were vaccinated with S19. Therefore, animals that were vaccinated with S19 will also test positive with serological tests. This limitation was not accounted for in the results nor was it clarified in the discussion.

Some animals included in the survey were vaccinated using reduced dose S19 via the conjunctival route (N = 28), however, these animals did not appear to alter the results/conclusions. Vaccination via the conjunctival route is assumed to produce a lower immune response than sub-cutaneous and reportedly less likely to interfere with serological tests (although the literature is unclear on this).

The following has been added to the discussion (lines 407:415):

*The S19 vaccine can interfere with serological tests (Avila-Calderón et al., 2013)Of the 28 animals vaccinated, 7 tested seropositive and it is unknown whether these were from infection (either prior to S19 conjunctival vaccination or because vaccination does not confer full protection). If these animals were removed from the analysis then the animal seroprevalence results would reduce from 15.1% to 14.7%, farm level seroprevalence. In addition, running logistic regression with vaccinated animals/farms removed led to the same variables being retained in the models. Therefore, these potential false positive results do not appear to be biasing the results.*

*Table 5:*

Is there an agreement between the two serological tests used for human samples? And what is the recommendation for future studies or to investigators that want to do this type of epidemiological study in other endemic countries?

Added to the discussion (lines 416:430). Methods are now more clearly described and section on study limitations added (line 393+) so other investigators can follow the study design and understand the drawbacks.

*In the current study and the study by Proch et al. (2018) a large proportion of persons seropositive by IgG ELISA did not test positive by RBT. The study in the general population also found poor agreement between the diagnostic test results in humans (Mangtani et al., 2020). This suggests there may be issues with the specificity of the ELISA used in these surveys, However, the results of the cross-sectional study and the parallel survey described in Mangtani et. al. (2020) show heightened exposure in persons in direct contact with livestock in the sampled dairy farms (9.7%) vs. general rural population (2.2%) and the risk factors identified correspond with the known biology of the disease, suggesting that misclassification due to low test performance may not have been an issue. Significant issues with the specificity of the iELISA would have likely resulted in more positive results in unexposed groups (false positives) than were observed. For example, of the 167 people who did not currently assist with calving and abortion, but had direct contact with livestock, only 5 (2.3%) were seropositive. Of these five people, three reported assisting with calving or drinking raw milk in the past. Compared to the superior situation for diagnosis in animals, there is a distinct lack of standardisation of serological reagents for diagnosis of human brucellosis.*

**Discussion:**

Line 4: *The study confirmed that brucellosis is endemic at high levels in cattle and buffalo of rural Ludhiana district in Punjab*. If both serological tests were used and most of the animals were positive, then yes. If not, a confirmatory test like culture or at least two serological tests are required to make a definite conclusion to avoid the bias of a high false-positive rate. Additionally, the limitation of the serological testing of animals on farms that vaccinated with S19 should be addressed and reflected in the results (please see the comment above).

The text has been changed throughout so the word ‘confirmed’ is not used. We agree with the reviewer that this term was confusing. The issue of S19 has been addressed in the results and discussion:

Paragraph 4 Line 1: *As people are primarily infected via animal sources, control in livestock (primarily ruminants) is key to reducing the public health impact of the disease.*

Results from this study indicate that human prevalence is only high in those in close contact with animals (e.g. farmers, livestock keepers etc.), hence the disease in this region appears to be more restricted to occupational exposure. While this study highlights the significance of occupational exposure to the disease, it doesn’t explain the prevalence in the general population especially because the disease does not appear to be transmitted through milk consumption which will pose a significant threat to the general population. This is because the authors reported that most people in the region in which the study was conducted consume milk that are either boiled or pasteurized, therefore, it seem that the general population that do not have any contact with animals or consume raw milk may not be at significant risk because humans are regarded as dead end hosts (human to human transmission of the brucellosis is rare). Therefore, the authors need to make stronger claims or speculate other ways in which the general population that have no contact whatsoever with animals may be at significant risk of *Brucella* infection.

When we say ‘general population survey’ we were referring to a survey where rural residents were randomly selected (these residents could still own cattle/work on a dairy farm). As 61% of rural households kept cows/buffalo in this study exposure was still a significant risk in this population. ‘General population’ does not exclude people in direct contact with animals – but these people comprise a smaller proportion of the sample as it was representative of the study area and not conducted in dairy farms.

This has been clarified as follows (lines: 354:358)

*In a ‘general population’ survey of brucellosis where households in the same villages as the current study were randomly sampled (regardless of cattle ownership), 2.2% of persons screened were seropositive for Brucella spp. (Mangtani et al., 2020). Risk factors for Brucella spp. seropositivity in this group were assisting with calving/abortion (61% of rural households kept cattle/buffalo) and consumption of goats’ milk.*

Reviewer #3: Overall this paper presents data from an interesting study in a context where data on brucellosis are limited. These data have definite value but are currently under-utilised and the presentation of the key findings from this work is not as clear as it should be.

Some updates to the structure of the article can greatly increase the focus and clarity of the work presented. There are also updates needed to the presentation of the statistical analyses

*We would like to thank the reviewer for recognising the value of the study! We greatly appreciate their efforts reviewing the manuscript and their valuable feedback. As a result of which we feel the manuscript has greatly improved.*

Are the objectives of the study clearly articulated with a clear testable hypothesis stated?

The objectives are given and are mostly clear. The third objective regarding human exposure risks could be more clearly stated (e.g. estimate prevalence and identify risk factors as for other populations rather than “exposure… was investigated” which is quite vague)

This has been edited (line 99:101):

*‘In addition, seroprevalence of Brucella spp. in persons in direct contact with these livestock was estimated and risk factor analysis performed.’*

There is quite a lot of content in the results and more so in the discussion that is not very clearly aligned to these objectives so more focus and structure in the reporting of these sections can help improve this.

We would like to thank the reviewer for this – we have cut-down sections of the results and discussion so that it is focussed and aligned to the objectives (see tracked changes version).

-Is the study design appropriate to address the stated objectives?
The design reported sounds appropriate but some details are missing.

For example,

Can you clarify/explain your dairy farm definition – were farms keeping 4 cows AND/OR 4 female buffalo included? – you specify “OR” only currently but I think included mostly farms keeping both species.

This has been reworded – farms with 4+ dairy animals (whether cows or buffalo) were included (line 105:106).

*‘For the purpose of the study, a ‘dairy farm’ was defined as any farm keeping at least four cows and/or female buffalo of reproductive age.’*

In the reporting of the multi stage selection can you reorder the content on Tehsil selection so that you explain ‘how’ selection was done at the beginning of section.
You report ‘(Jagraon, Payal and Samrala)’ here without any explanation of what these are.

This has been reworded (lines 123:131)

*In the first stage of sampling, four out of seven Community Development Blocks (CD block) (administrative division) of Ludhiana district were selected to restrict location for practical purposes. East Ludhiana has the largest human population (Census of India, 2011), therefore this CD block was included and every other CD block was sampled working anti-clockwise in a systematic manner (Jagraon, Payal and Samrala). Within these CD blocks, 60 villages were randomly selected using sampling probability proportional to human size (Census of India, 2011).*

The order of these section is not very clear. The sample size content could be moved to the beginning or end but appearing in the middle of the description of selection steps is quite confusing. Can you explain how you chose the value of 6% precision for this analysis as that’s not a standard default value?

The sample size content has been moved to the beginning of the section. The following has been added to the text (lines: 116: 118)

*Different precision values were trialled and 6% was selected as a trade-off between estimate accuracy and number of herds that could be sampled given available resources.*

Can you give an indication of the denominator in each step? E.g. is 8 dairy farms per village a large of small % of the total? You say ‘up to eight dairy farms” were selected. When and why was this less than 8?

It was less than eight if eight eligible dairy farms could not be identified to participate in the survey in the selected village, this has been made clearer.

The average population of a village in the study area is approximately 1500 people with an average household size of 5.2 persons (Census of India, 2011). From the general population survey (random sample of households regardless of cattle ownership), 61.0% of families reported keeping cattle and 45% met the criteria for eligibility in the dairy farm survey (4+ adult female/buffalo). Therefore, it is estimated that an average village would contain 72 herds. However, from our experience this was highly variable.

Additional text added (lines 129:138):

*In order to meet the required sample size, eight dairy farms per village were selected using simple random sampling. In an average village, eight farms were estimated to comprise around 10% of the total dairy farms (Census of India, 2011; Mangtani et al., 2020). Lists obtained from the designated veterinary officer or pharmacist of each village were used as a sampling frame. If eight of fewer eligible farms were present in the study area then all farms were approached for inclusion in the study.*

If the farm had > 9 animals (NB – is this > 9 animals total or > 9 lactating females?) you report that a systematic sample was taken. How many animals is this? What criteria were sampling decisions made on? Was this really systematic sampling or convenience? Either is defendable – but it needs to be clear. The relevance of the statement re tethering is not clear.

There was a grammatical error in the sentence which has now been corrected (lines 134:138):

*‘If the dairy farm had up to nine lactating animals then all lactating animals were sampled, where possible. If the dairy farm had more than nine animals then a systematic sample of* ***nine*** *animals was taken.’*

In this case, sampling was really systematic - the relevance of tethering is that the livestock are not grazing, mobile and are mostly tethered in a line at a feeding trough. This facilitates systematic sampling as the animals are lined up and not moving around – whereas in a field/pastoralist setting it is more difficult to count every nth animal. Although this sentence has been deleted as probably unnecessary detail.

Can you give more rationale for the repeat testing approaches used and present the data on test repeatability for the different populations and tests in the results. Please report the results of the ‘validation’ of RBT antigens also – this can be in the SI but are important data to make accessible for evaluation.

**Livestock samples:**

The rationale of testing the samples twice with the same is ELISA was to increase the specificity given the brucellosis was endemic in the study area, and to validate borderline results. Very occasionally if cream is added to the wells this can reduce the specificity (potentially resulting in false positive). Although cream was avoided for all testing, if a sample gave a positive-results, extra care was taken to avoid the cream on repeat testing.

In terms of repeatability of the milk ELISA 209, negative results were retested: 207 were negative and 2 became positive. The ODs of these samples on the first test were 97% and 64% of the cut-off. Of the 312 positive results retested, 38 became negative. The majority were borderline and only 7 of these were had OD’s higher than the medium positive in the first test). Therefore, the milk ELISA had good repeatability. There is also potential for human error with re-testing samples and recording the correct ID’s for discordant samples.

For the reviewer’s information, another aim of the project was to assess correlation serum was collected from a sub-set of these livestock and screened using RBT (results being prepared for publication elsewhere). The milk ELISA and RBT showed very high correlation – results shown here (Inc are those samples producing discordant results with milk ELISA):

|  | | **RBT** | |
| --- | --- | --- | --- |
|  |  | **-** | **+** |
| **sLPS iELSA (milk)** | **-** | 388 | 0 |
|  | **inc** | 1 | 7 |
|  | **+** | 2 | 79 |

Added to the text (lines 248:251)

*‘Of the 1802 milk sample collected from cattle and buffalo, 312 gave a positive result test and 272 produced two consecutive positive results using the commercial IgG ELISA (BRUCELISA®). Therefore, individual livestock seroprevalence was estimated to be 15.1% (95% CI: 15.9 to 19.8).’*

**Validation of RBT (lines 188:189)**

*‘A subset of samples (10 negatives, 7 positives) were also screened with antigen from the Animal and Plant Health Agency (APHA), UK which is an FAO/OIE/WHO International Reference Laboratory for Brucellosis for quality assurance. These samples showed perfect correlation.’*

The level of detail given for different tests is variable – e.g. you report the cu-off rationale and rules for the BRUCELISA but not the DEMEDITEC test so this should be standardised (and explain how the kit defines inconclusives)

Added the following (lines 190:197):

*All serum samples were then tested for IgG antibodies for Brucella spp. using commercial ELISA kits supplied by DEMEDITEC Diagnostics GmbH, Kiel, Germany. As per the manufacturer’s standard kit protocol, optical densities (ODs) were read at 450nm. For each plate, samples were also interpreted as positive if ODs were >20% over the manufacturer’s cut-off standard, negative as <20% under the cut-off, and inconclusive if in-between. All inconclusive ELISA samples were classified as negative for the purposes of this study. Both serological tests detect antibodies against the smooth lipopolysaccharide shared between the smooth strains of Brucella spp. (B. abortus, B. melitensis and B. suis).*

Please give more detail on the performance, results and guidance based on any SATs on human samples.

Added the following:

**Methods (lines 203:207)**

*Persons with a SAT titre of 1/160 International Units (IU) or higher received a phone-call from the principal investigator at GADVASU and were referred to a clinician at GADVASU who works with farmers and veterinarians to diagnose and treat brucellosis in the study area for further follow-up if they agreed.*

**Results (lines 300:303)**

*Of the 57 people that were classified as seropositive, 13 (22.8%) produced SAT titres of 1/160 or higher. All of these were RBT positive, however, only 2 mentioned symptoms in the last 12 months.*

The exact questions asked of respondents and the period of reference for the questions about risk behaviours should be included in the methods (the survey tool could be included as an SI file).

Noted – the questionnaire for humans has been included as a supplementary file.

-Is the population clearly described and appropriate for the hypothesis being tested?

The rationale for combining cattle and buffalo in several design and results reporting steps is not clear. What was the prevalence in each species? In the reporting of the model results the presence of different species and herd composition appear to be important but the detail on these metrics is not given or discussed very clearly.

The prevalence in each species is now reported in the text, in addition interactions with species in the multivariate model were tested for and the results section has been revised to discuss the results more clearly. The majority of herds were mixed (58.8%) therefore it did not make sense to perform separate analysis for cow and buffalo herds, although species was investigated in herd and individual models (lines 251:254).

*Overall, 96 (11.9%) buffalo and 174 (17.9%) milk samples from cows tested positive.*

The composition of the human population reported on should be clearer. Can you give a clearer description of how this population was defined? Did you record data on the proportion of eligible individuals that ‘accepted’ the offer of screening? If not, can you estimate this? Assuming < 100% uptake what biases might this introduce and how does that affect your interpretation of the findings?

A study limitations section has now been added (line 393+)

In all of the tables and figures please include ensure that the population (animals, people or herds/farms) is clearly and simply stated each time – e.g. tables S5 and S6 are presumably about serostatus in humans but that isn’t stated.

We would like to thank the reviewer for pointing this out and this has been done.

-Is the sample size sufficient to ensure adequate power to address the hypothesis being tested?

This calculation can be more clearly described. The content in the main paper is not very informative and I’d recommend stating the values currently reported in the SI in the text here so that all of the assumptions of the calculation are given up front. Please include a statement in the text of the sample size that the calculation indicated required (and then in the results, explicit statement that this was achieved)

This has been added as follows (line 104:122):

*Between June 2015 and September 2017, a cross-sectional study of dairy farms was conducted using a multi-stage sampling strategy. For the purpose of the study, a ‘dairy farm’ was defined as any farm keeping at least four cows and/or female buffalo of reproductive age. Herds were classified as positive if at least one animal tested seropositive. Assuming an average of four lactating animals sampled per herd and an individual test specificity of 99% (Nielsen et al., 2001, 1996; Vanzini et al., 2001, 1998), herd specificity (HSp) was estimated to be 96%. Values of HSe (likelihood of correctly classifying an infected herd) varied depending on herd size, number of lactating animals sampled and within-farm prevalence. Different combinations of these values were explored in order to achieve a desired HSe of 70% (EpiTools, 2009).Given a design prevalence of 10% (the minimum within-farm prevalence to detect), sampling a total of nine animals per herd would result in HSe values above 70% given the values presented it Table X. A sample size of 384 herds would allow herd-level prevalence to be estimated with a 6% precision and 95% confidence. This was multiplied by an assumed design effect of 1.07, estimated from previous surveys, to account for correlation of herds within villages to give a final sampling target of 411 dairy farms.*

Table 1 Values used to calculate sample size in order to estimate farm-level prevalence

| **Variable** | **Value** | **Ref** |
| --- | --- | --- |
| Herd size | 5 – 20 | Pilot study |
| No. lactating animals sampled | 1 – 9 | Inputted value |
| Design prevalence (min) | 0.2 | Inputted value |
| Individual test sensitivity | 0.98 |  |
| Individual test specificity | 0.99 |  |
| Calculated herd sensitivity (min) | 0.72 | <http://epitools.ausvet.com.au/content.php?page=HerdSens4> |
| Calculated herd specificity | 0.95 |  |
| Expected herd-prevalence | 0.25 | Expert opinion |
| Desired Precision (confidence) | 0.06 (0.95) | Inputted value |
| No. farms in study area | 10,000 | Calculated from Census of India. 2011 |
| Calculated sample size – unadjusted for clustering | 384 | Purposefully-designed spreadsheet |
| Design effect | 1.07 | Assumed from previous studies |
| Total herds – adjusted for clustering | 411 |  |

-Were correct statistical analysis used to support conclusions?

The description and presentation of many of the stats and findings is often very descriptive and quite repetitive. You also report GLMM analyses that sound entirely appropriate for this dataset but then revert back to presentation and interpretation of raw data over the model outputs.

This section would be clearer if reordered. Start with clear description of the populations and outcomes modelled. I think that a common approach was taken to model development for the 3 different populations. If so, compile all of the descriptions in one place and state this clearly. The description of the forward model building is not very clear. What order were variables evaluated and added in? Can you include references to support the decisions made based on coef and SE changes etc.

The methods have been revised extensively based on the reviewers’ comments (see responses to comments below and track changes).

You state that variables with a p<0.2 in univariable analyses were evaluated in multivariable models but its not clear what test the p value(s) mentioned are related to? If one level of a multifactor variable has a p for the coef estimate > 0.2 does that variable go through? Why were LRTs not used to evaluate the contribution to model fit of the variables as a whole?.

This has been clarified for the univariate analysis (lines 233:234):

*‘Variables where one or more categories were associated with Brucella spp. seropositivity with a P-value ≤ 0.2 were retained for further multivariate analysis.’*

Multivariate analysis (lines 227:231):

*‘A manual forward step-wise procedure was used to build the models with variables with the lowest P-values entered into the model first. Upon addition of each variable, likelihood ratio tests were performed comparing the fit of the new model with the previous and to compare whether model fit was better when linear variables were kept as linear exposures or categorised.'*

I think all models are logistic regressions with binomial errors but this is not clearly described (except in Table 3 legend).

This is correct and has been added in the methods.

Many apparently continuous variables are categorised for model analyses and its not clear why? E.g. number of animals in different groups. Please explain or update as this seems to be an inefficient use of data. This also appears to change – in Table 3 the number of cows variable appears to be continuous?

The univariate tables were also meant to describe the population of interest, hence, why they have been categorised in the univariate analysis (lines 229:231).

*Upon addition of each variable, likelihood ratio tests were performed comparing the fit of the new model with the previous and to compare whether model fit was better when linear variables were kept as linear exposures or categorised.'*

Can you explain justification of manual calculation of ICC metrics rather than reporting and using metrics derived from the random effects estimated in the models. Even if not used this way, all summaries of the GLMMs should include summary of the random effects estimated.

This has been corrected and GLMM ICC’s are now presented (there were some errors in the previous calculations, thanks to the reviewer for pointing this out):

Line 270:272

*The adjusted ICC of the farm model was 0.11, suggesting that 11% of the variance can be accounted for by the clustering of people within village.*

Line 285:286

*The adjusted ICC for individual livestock was 0.164 for village and 0.005 for farm.*

Line 315:317

*The adjusted ICC of the model for persons in direct contact with livestock was 0.204.*


Can you explain why the farm/herd RE was not included in the model of human serostatus data? Was this evaluated? This is presumably as you had many farms with 1 observation but should be stated clearly.

Exactly – this has been stated clearly (lines 219:222).

*‘Farm was not included as a random effect for the model investigating risk factors in people in direct contact with livestock as only one person was tested in 24.3% of farms.’*

-Are there concerns about ethical or regulatory requirements being met?

There is a mention in the methods that human serum agglutination tests were performed but no results are given on this. Were any presumptive cases identified? What case definition was used for this step and what procedures were followed for data sharing and clinical advice for individuals who were tested – and then received positive or negative results?

Lines 203:207

*‘Persons with a SAT titre of 1/160 or higher received a phone-call from the principal investigator and were referred to a clinician at GADVASU who works with farmers and veterinarians to diagnose and treat brucellosis in the study area for further follow-up if they agreed.’*

Does the analysis presented match the analysis plan?

The ordering of the results could be more clearly mapped to the study objectives – e.g. consistent ordering of animals, herds and humans. The livestock results sections opens with a description of milk consumption patterns which aren’t really mentioned before this point. Similar the dairy farms section is focused on the small n farms that kept goats. This is a confusing point to lead with as its not focused on any of the stated objectives, the data are very sparse and none of the results presented are informed by the model analyses performed. Several associations are reported without an explicit statement of (or reference to the table) the results from the analyses presented that support each statement.

Originally the main descriptive data was presented in the tables and the text contained additional descriptives of the study population that we felt the readers may find interesting. However, we agree with the reviewer and have revised the results section and cut-down descriptions of variables that are not included in the risk factor analysis in order to those included in the analysis (the lines the reviewer refers to have been removed).

Its not clear why the data for cattle and buffalo are reported together vs by species.

Many of the herds were mixed, therefore, for herd-level analysis they could not be separated. However, prevalence is now reported for each species. Also, important production parameters such as parity, age of the animals in the herd etc. did not appear to differ between species. Although cows were more likely to be infected, the measure of effect was not very high (OR = 1.75) the and they could be additional variables related to management that were confounding this association.

Much of the reporting of the univariable analyses can be dropped so that the results are focused on the multivariable model findings. All of the univariable results are given in the tables (I think) but these are not consistently presented. Some include indications of the variables evaluated in multivariable models but not all. The explanation of many variables is unclear – does Buffalo Yes/No mean indicator of species presence on the farm?

The Univariate results have been removed from the text and the tables explained more clearly (we can also remove univariate analysis tables to supplementary information if the reviewer feels that is clearer).

Variables taken through to the multivariate models are marked in the tables and explained more clearly in the causal diagram (Figure 1). The labelling of variables in Tables has been revised.

There are several cases where a set of correlated variables are evaluated and its not clear how decisions were made on which variable to carry through to multivariable analysis? E.g. were model presence of buffalo and herd type fitted in the same models?

Where collinearity has been observed the rationale for the retained variable is now given more clearly:

Lines 317:318:

*Gender was not included as a potential confounder as there was collinearity between gender and the exposures due to gender roles within the farm.*

Lines 321:322

*Assisting with calving and assisting with abortion showed collinearity and these variables were combined in to a new variable (“assisting with calving and/or abortion in the last 12 months”).*

Lines 283:285

*Species and breed exhibited collinearity so only one was retained in the model. It was decided to keep species as there was some heterogeneity with how breed was recorded and some categories had few observations.*

Lines 321:323

*The results of the multivariate model are presented in Table 8. Assisting with calving and assisting with abortion were combined in to a new variable (“assisting with calving and/or abortion in the last 12 months”) as GLMM did not converge when included separately in the final model.*

-Are the results clearly and completely presented?

The levels of many factors are not really explained. Its also important to explain clearly how some of the factor levels are defined. Its not clear from the info given that the decision to combine Never and past and compare with “Yes” is a justifiable choice. What are the time periods for each level? Why

There were too few observations for ‘past’ in the dataset for it to remain a standalone category. Although we agree with the reviewer that collapsing of never and past is not ideal, the proportions seropositive for ‘past’ are much closer to ‘never’ than ‘current’. Therefore, we felt by combining ‘past’ with ‘current’ we would make incorrect conclusions.

The time periods are now specified (in the last 12 months) and the tables have been labelled more clearly.

Do you need to present both model 6 and 7? Why is age retained in the model?

Only the final multivariate model is presented – age was included as an *a priori* confounder.

In some of the results test sections the results focused on are not very obviously focused on the objectives of the paper and involve quite a lot of anecdotal description of data, to suggest associations that were either not statistically evaluated or are not support by the models presented – e.g. symptom reporting in occupationally exposed period. Given the very low diagnostic value of clinical signs, use of serostatus vs clinical illness as the outcome, long persistence of Ab and retrospective nature of the survey there is no very strong reason to evaluate evidence of any association. These data are not mentioned elsewhere in the methods or results.

We agree with the reviewer and had not investigated statistical relationships between symptoms and test results for this reason. Reference to the symptom reporting is now deleted from the results.

You describe a slight increase of seroprevalence with age based on the figure but as the models indicate no association I don’t its justifiable to present this as an increase with age.

This has now been removed, to focus on key results this figure has been deleted.

Quite a lot of the descriptive text on e.g. milk consumption practices could be replaced by referring to the detail on these data (and the model evaluations performed) in a table (s6) rather than brief text summary.

The text has now been cut-down substantially and the table referred to.

The interpretation of the data on risk factors for human exposure is complicated by the lack of clarity on the population sampled and the way the variables were evaluated. The rationale for the descriptive analysis of association between different explanatory variables is not clear. Why weren’t these effects evaluated using interaction terms? The nature of the comparator population in reporting of the increased risk in people with occupational exposure to seropositive animals is not clear. Its also unclear why a new model is used to evaluate this variable/interaction vs including the interaction in the full model for this population?

Interaction was tested for, however, the very few observations in some categories (positive people that did not assist with calving/abortion in both positive and negative farms) was insufficient for inclusion of an interaction term between these variables and the model did not converge. Therefore, a new variable was created combining people that ‘do not assist with calving/abortion’ into one category. However, only the final multivariate model is now included in the text:

Lines 321:333

*The results of the multivariate model are presented in Table 8. Assisting with calving and assisting with abortion were combined in to a new variable (“assisting with calving and/or abortion in the last 12 months”) as GLMM did not converge when included separately in the final model. In the univariate analysis, people in direct contact with seropositive cattle and buffalo had 1.85 (0.98 to 3.50) times the odds of testing seropositive for Brucella spp. compared to those that did not. A biologically plausible interaction between farm status and association between assisting with calving/abortion and brucellosis status was investigated. However, only 5 people (5.7%) that did not assist with calving/abortion were seropositive (1 from a seropositive farm and 1 from a seronegative farm). As there were two few observations to include an interaction term between these variables, a new variable with three categories was created: i) assisted with calving and/or abortion in the last 12 months and from a farm classified as seropositive, ii) assisted with calving and/or abortion in the last 12 months and from a farm classified as seronegative and iii) have not assisted with calving or abortion in the past 12 months.*

A causal diagram is now included (Figure 1) . The methods have been revised based on previous comments by the reviewer so that sampling methods are more transparent.


-Are the figures (Tables, Images) of sufficient quality for clarity?

In the first figure can you update the legend to explain the overlap shading or update the plot so that the data for the sexes are presented adjacent but not overlapping.

This Figure is now deleted (see previous comment)

In the tables, more detail on the variables and levels presented are needed.
What is “LR”?

This was large ruminants but it has now been deleted.

Reviewer #3: There is quite a lot of content in the discussion about a sister paper that is not yet published. Its not really appropriate either present findings from another study here or to include reference to the other study until the other paper can be cited.

This study has now been published and can now be cited.

-Are the conclusions supported by the data presented?

The speculation about infecting species of Brucella is not supported by any of the data presented in this paper and I would not include this for that reason

This has been removed.

Some of the points about relative importance of different routes of transmission are interesting but don’t make as good use of the available data as is possible to support these points.

This has been revised (line 366:384).

*The primary risk factor for testing seropositive for Brucella spp. in this survey was assisting with calving or abortion; cattle and buffalo infected with Brucella spp. excrete high concentrations of the organism in placental membranes and aborted foetuses. This effect depended on farms status; people that assisted with calving/abortion on a farm with seronegative livestock and people that assisted with calving/abortion on a farm with seronegative livestock and people that did not assist with calving/abortion had 0.35 (95% CI: 0.17 to 7.1) and 0.21 (0.09 to 0.46) times the odds of testing seropositive compared to persons assisting with calving/abortion in a seropositive farm in the last 12 months. This strongly supports the existence of a relationship between assisting with calving and abortion of seropositive cattle and infection.*

*The results of this survey, supported by the parallel survey in the general population suggest that there is limited exposure of Brucella spp. to people via milk and dairy products produced by large ruminants. This is likely due to the common practice of always boiling milk before consumption (85.1% in this survey) or as part of processing it into other dairy products.*

*Given that approximately 20% of those that assisted with calving or abortion in a positive farm had evidence of exposure to Brucella spp., it is likely that transmission via this route is more important than the foodborne route in the study population. India has the largest bovine and second largest human population in the world. Although these results cannot be extrapolated, if the situation is similar in other Indian states producing high volumes of bovine milk, transmission of B. abortus via direct contact may be responsible for a significant burden of brucellosis infections in people.*

The description of the counterintuitive finding is a little speculative. The argument that people might take more precautions during parturition if aware that there was brucellosis in their herd is somewhat undermined by the low levels of awareness of the disease that is presented elsewhere?

The low level of awareness refers to the disease in humans (i.e. people are not aware of it as a zoonosis). Dairy farmers are more aware of the disease in livestock (although we agree the point is speculative and have removed).

The content on possible control, surveillance and transmission prevention strategies is interesting and important but is currently mentioned in a few different places and is not really linked to any of the data presented or clearly described in the context of options under consideration in this setting. What control options might be feasible in this context? How do the data presented here help guide thinking about these options?

Implications of these findings on control, surveillance and transmission has been revisited (lines 432:467)

*The best strategy for preventing introduction into negative dairy farms would be to screen new purchases, however, only four livestock owners reported doing this. The feasibility of this strategy depends on availability of screening tests for brucellosis at village level and farmers’ capacity to afford this. According to this survey around a third of dairy farms already had at least positive animal in their herd, therefore, management strategies to reduce within-herd transmission of Brucella spp., particularly when animals are calving may be more effective. Although the majority of farms did report separating livestock at the time of calving (62.5%), they are often still in close proximity to the rest of the herd and are returned to the herd very soon after.*

*At the time of this study a programme termed ‘Brucella free village’ was launched by the Department of Biotechnology with the mandate to “control and eradicate brucellosis in animals through stamping out of positive animals, vaccination of animals and maintaining sanitary conditions”. The initiative is planned to be piloted across 50 villages in 10 states, including Punjab. The programme is very comprehensive planning to combine intensive screening, segregation of positives and vaccination. Although this will lower disease frequency in the selected villages there are concerns with the feasibility of upscaling this programme. The majority of the villages in this study would be classified as ‘highly endemic’ according to this programme’s classification (prevalence >5%). The programme plans to isolate positive cows outside the village and slaughter positive buffalo. Given that almost 1 in 6 animals were seropositive in the cross-sectional survey, this strategy would require huge resources in this setting and may not be feasible unless prevalence was first sustainably lowered through methods such as vaccination.*

*A workshop was held at PGIMER campus on 21^`^November 2016 between State-level livestock, public health and food-safety actors around the theme of brucellosis control. The main objectives of the workshop were to ascertain what is being done to control brucellosis in Punjab State, identify opportunities to improve control, share project results with stakeholders and facilitate intersectoral communication. Nineteen participants attended the meeting, representing the main institutions involved in zoonosis control in Punjab, including Departments of Animal Husbandry, Dairy Development and Health and the Food and Drug Administration. In addition, public and animal health researchers, clinicians and representatives from the private sector (cooperatives and milk union) were present. Participants agreed that brucellosis was an important animal and public health disease in the state. However, despite vaccination policies in place low coverage is achieved, this is supported by the findings of this study. Stakeholders attributed low coverage lack of vaccine availability and safety concerns of veterinarians and farmers. Therefore, these issues would need to be addressed before any vaccination campaigns are implemented.*

There is quite a lot of narrative content presented in this section describing findings from other studies (e.g. on PPE) that don’t currently add a lot to the interpretation of the findings from this study.

Good point – this section is now been reduced.

-Are the limitations of analysis clearly described?

There is not an explicit section on study limitations

This has now been added (line 393+)

-Do the authors discuss how these data can be helpful to advance our understanding of the topic under study?

-Is public health relevance addressed?

The public health relevance of the findings is mentioned but the content is quite vague and non-specific. The general points about the challenges of vaccination are not really the findings of this study. Can you include more focused content on what the findings of this study tell us and how they could be used to guide or focus next steps research or pollcy?

The discussion has been revised extensively and more focussed to what we conclude on the basis of the results found and the implications/limitations of the study (lines). We hope the reviewer finds the discussion improved (lines 463:483)

*As people are primarily infected via animal sources, control in livestock (primarily ruminants) is key to reducing the public health impact of the disease. However, whilst the seroprevalence remains high, it is recommended that the Dept. of Animal Husbandry works with health authorities to design public health campaigns which could reduce the risk of human exposure to brucellosis. Most people sampled said they were not aware of brucellosis as a disease in humans (93.4%). Further, through discussions during fieldwork and the stakeholder workshop, it seems that rural healthcare workers are also unaware of the disease. Therefore, it is likely that brucellosis goes unrecognised or misdiagnosed at the level of primary healthcare centres. Awareness was created in the sampled villages, however, there is a need to disseminate this information further. The issue of human diagnostics was also discussed during the workshop and clinicians raised highlighted the need to include brucellosis in the guidelines for investigating pyrexia of unknown. A technical expert group on human brucellosis was established as an output from the stakeholder workshop. This group is developing best practice guidelines for treatment and diagnosis and fostering awareness in the public health sector. Further as a result of this survey, samples are being sent to GADVASU from tertiary hospitals for screening.*

Reviewer #3: Please include page and line numbers for future submissions as that greatly improves the review process

These have been added – apologies for omission from first version.

There are several places where the terminology is inconsistent which creates ambiguity and confusion. Try to use a stripped back set of terms consistently - e.g. herds vs dairy farms vs farms, the descriptions of the various human populations need clarification throughout (people present ‘involved with husbandry’, household members, family members, farm workers, occupationally exposed group etc are used at various points and its not clear how these all relate to each other).

The terminology has been altered to be consistent – the human population under study is now described as ‘persons in direct contact’ with the livestock in the dairy farms under study. This group comprises; family members, farms workers and farm owners.

The study unit for livestock is now described as ‘dairy farms’ throughout, farm-level prevalence/risk factor analysis is used. Although ‘herd’ is occasionally used to refer specifically to the group of animals within the farm e.g. the sentence below where the use of ‘dairy farm’ would cause some ambiguity as to whether we meant proximity to the farm premises rather than the animal within.

*“In addition, although the majority of farms did report separating livestock at the time of calving (62.5%), they are often still in close proximity to the rest of the herd.”*

There are acronyms that need to be checked for full explanation at first use also.

These have been checked

--------------------
